# Timing of HIV testing among pregnant and breastfeeding women and risk of mother‐to‐child HIV transmission in Malawi: a sampling‐based cohort study

**DOI:** 10.1002/jia2.25687

**Published:** 2021-03-21

**Authors:** Maganizo B Chagomerana, Jessie K Edwards, Lauren C Zalla, Nicole B Carbone, Godfrey T Banda, Innocent A Mofolo, Mina C Hosseinipour, Michael E Herce

**Affiliations:** ^1^ University of North Carolina Project/Malawi Lilongwe Malawi; ^2^ Department of Medicine University of North Carolina School of Medicine Chapel Hill NC USA; ^3^ Department of Epidemiology Gillings School of Global Public Health University of North Carolina at Chapel Hill Chapel Hill NC USA

**Keywords:** antiretroviral therapy, mother‐to‐child transmission, HIV, Option B+, PMTCT, viral suppression

## Abstract

**Introduction:**

Pregnant women living with HIV can achieve viral suppression and prevent HIV mother‐to‐child transmission (MTCT) with timely HIV testing and early ART initiation and maintenance. Although it is recommended that pregnant women undergo HIV testing early in antenatal care in Malawi, many women test positive during breastfeeding because they did not have their HIV status ascertained during pregnancy, or they tested negative during pregnancy but seroconverted postpartum. We sought to estimate the association between the timing of last positive HIV test (during pregnancy vs. breastfeeding) and outcomes of maternal viral suppression and MTCT in Malawi’s PMTCT programme.

**Methods:**

We conducted a two‐stage cohort study among mother–infant pairs in 30 randomly selected high‐volume health facilities across five nationally representative districts of Malawi between 1 July 2016 and 30 June 2017. Log‐binomial regression was used to estimate prevalence ratios (PR) and risk ratios (RR) for associations between timing of last positive HIV test (i.e. breastfeeding vs. pregnancy) and maternal viral suppression and MTCT, controlling for confounding using inverse probability weighting.

**Results:**

Of 822 mother–infant pairs who had available information on the timing of the last positive HIV test, 102 mothers (12.4%) had their last positive test during breastfeeding. Women who lived one to two hours (PR = 2.15; 95% CI: 1.29 to 3.58) or >2 hours (PR = 2.36; 95% CI: 1.37 to 4.10) travel time to the nearest health facility were more likely to have had their last positive HIV test during breastfeeding compared to women living <1 hour travel time to the nearest health facility. The risk of unsuppressed VL did not differ between women who had their last positive HIV test during breastfeeding versus pregnancy (adjusted RR [aRR] = 0.87; 95% CI: 0.48 to 1.57). MTCT risk was higher among women who had their last positive HIV test during breastfeeding compared to women who had it during pregnancy (aRR = 6.57; 95% CI: 3.37 to 12.81).

**Conclusions:**

MTCT in Malawi occurred disproportionately among women with a last positive HIV test during breastfeeding. Testing delayed until the postpartum period may lead to higher MTCT. To optimize maternal and child health outcomes, PMTCT programmes should focus on early ART initiation and providing targeted testing, prevention, treatment and support to breastfeeding women.

## 1 INTRODUCTION

Antiretroviral therapy (ART) for HIV‐positive pregnant and breastfeeding women (PBFW) has proved to be life‐saving for both mother and infant. As a treatment for HIV, ART improves maternal health and survival [1]. As a prevention tool, ART during pregnancy and breastfeeding has drastically reduced mother‐to‐child transmission (MTCT) [2], giving hope for global elimination of new paediatric HIV infections. In settings with high HIV prevalence, these benefits have become more evident since introduction of the WHO‐recommended Option B+ and “treat all” strategies [3, 4], enabling lifelong ART for PBFW regardless of CD4+ count or WHO clinical stage. To ensure that all HIV‐positive PBFW in Malawi are on ART, PBFW who have unknown HIV status or who tested HIV negative previously are offered HIV testing during antenatal care (ANC), in maternity (labour and postnatal wards), and during the post‐natal period in child immunization clinics [5]. PBFW who test positive on a confirmatory rapid test are started on ART immediately [5].

Early ART initiation, adherence, and maintenance during pregnancy and the breastfeeding period are necessary to prevent MTCT. Women who initiate ART during breastfeeding, particularly after a delay in HIV diagnosis, experience a higher MTCT risk compared to those initiating ART promptly during pregnancy due to uncontrolled viraemia or unidentified seroconversion during pregnancy or breastfeeding [6]. Newly seroconverted women with acute HIV have high levels of viraemia [7, 8], and are more likely to experience MTCT than women with established HIV infection [9, 10, 11]. Although the majority of HIV‐positive PBFW in the Malawi Option B+ programme start ART during pregnancy, an estimated 10% to 15% start ART during breastfeeding [12]. HIV‐positive PBFW who initiate ART during breastfeeding may include: (1) women with established HIV infection who never tested during pregnancy and delivered at home; (2) women with established HIV infection who tested positive during ANC but never initiated ART; (3) women who re‐initiated ART during breastfeeding after treatment interruption and (4) women who seroconverted late in pregnancy or during breastfeeding.

Despite being an especially high‐risk group, limited data from high‐burden countries fully characterize the demographic and social characteristics of mothers found to be HIV positive during breastfeeding in the current treat all era. Moreover, in Malawi, relatively little is known about the comparative risk of viral suppression and MTCT for mothers identified with HIV infection during breastfeeding versus those found HIV positive during pregnancy. In this paper, we use data from a sampling‐based cohort study done in five Malawi districts to address these pressing evidence gaps. Specifically, we assess risk factors for having a last positive HIV test and initiating ART during breastfeeding, and estimate the association between timing of the last positive HIV test and viral load (VL) suppression, as well as MTCT.

## 2 METHODS

### 2.1 Study design

Our study used an observational cohort design with two stages. In the first stage, we reviewed all available medical records to construct a “full cohort” of all women living with HIV eligible for the national prevention of mother‐to‐child HIV transmission (PMTCT) programme in randomly selected high‐volume facilities (i.e. “sites”) in five Malawi districts between 1 July 2016 and 30 June 2017. The five districts—Lilongwe, Mzimba North, Mzimba South, Salima and Zomba districts—were purposively selected to include urban, peri‐urban, and rural catchment areas and to represent a diversity of non‐governmental implementing partners supporting the national PMTCT programme. We chose the period of July 2016 to June 2017 to align with rollout of national universal “test and treat” (UTT) guidelines that reached maturation (i.e. guidelines issued, healthcare providers trained, and programme started in most facilities) around July 1, 2016. In early 2016, Malawi adopted UTT as policy, which extended its successful Option B+ strategy, first introduced in 2011, to include all people living with HIV (PLHIV) who could now initiate ART regardless of CD4+ count or WHO clinical stage. In the second stage, we randomly selected a sample of at least 30 women living with HIV per site from a fully enumerated list of all women included in stage one, to create a “nested cohort” of women who then underwent a field survey to collect additional questionnaire and biomarker data. In this way, the second stage of our two‐stage design enabled us to enrich the routine medical record data from the first stage with prospectively collected data on health behaviours and biological outcome measures of maternal VL and infant HIV sero‐status. All eligible women provided informed consent to participate.

The study was approved by the Malawi National Health Sciences Research Committee (UNCPM #21708), and the institutional review boards of the University of North Carolina (#17‐1114), Brigham & Women’s Hospital (Reliance Agreement #17‐1114), and James Cook University (HREC #1812).

### 2.2 Participants

Mother–infant pairs in which the mother satisfied the following criteria were eligible for nested cohort enrolment: newly diagnosed or having documented evidence of HIV infection between 1 July 2016 and 30 June 2017 in selected health facilities in the five study districts; documented referral to, or enrolment in, Malawi’s PMTCT/ART programme; pregnant (at any gestational age) or breastfeeding at the time of their last positive HIV test; and ≥16 years old at the time of PMTCT referral/enrolment (includes adults and emancipated minors according to Malawi law).

### 2.3 Data collection and management

During the first stage of the study, we abstracted existing clinical and demographic data from routine MOH treatment cards and registers and from electronic medical records. Abstracted data included: HIV testing history; 6‐, 12‐, and 24‐month ART programme status classification (i.e. alive in care, transferred out, stopped ART, died, and lost to follow‐up); 6‐ and 24‐month maternal VL (timing per national guidelines); exposed‐infant first HIV‐1 DNA PCR result (from six weeks of age per national guidelines) [5]; 12‐ and 24‐month infants exposed to HIV (HEI) vital status (including death) and 12‐ and 24‐month HEI HIV sero‐status.

To establish the nested cohort, we randomly selected participants from an enumerated register of all mother–infant pairs who met study eligibility criteria based on a sampling fraction between 75% and 100%. Study and MOH staff used detailed locator information available at each clinic to contact sampled participants at clinic visits and through community outreach, telephone calls, and/or in‐person tracing. All women who consented to participate in the nested cohort completed a structured questionnaire to provide information about demographics, pregnancy and breastfeeding history, their sexual partner(s), and their experiences with MOH PMTCT services and community‐facility linkage (CFL) model services. The CFL models examined included Expert Clients, Community Health Workers, and Mentor Mothers, and have been described elsewhere [13]. If there were no VL or HIV test results documented in the routine medical record or if the documented results were for tests done ≥90 days before study data collection, a one‐time blood sample was collected from both mother (dried blood sample [DBS]) and infant (rapid HIV‐1/2 antibody testing or DBS, depending on age as per national testing guidelines) for VL testing and HIV status ascertainment respectively.

### 2.4 Exposure ascertainment and definition

Our main exposure was the timing of the last positive HIV test categorized as during either pregnancy or breastfeeding. In the treat all era, in addition to providing information about HIV diagnosis, the timing of the last positive HIV test can serve as a proxy for timing of ART initiation since treatment recommendations call for rapid, usually same‐day, ART start. In this analysis, we focused on the timing of the last positive HIV test to create a proxy for the timing of ART initiation for the index pregnancy because some women may have been diagnosed with HIV previously, but only started ART (or restarted ART following treatment interruption) after testing positive again during ANC, in maternity, or child immunization clinics [5]. We conducted a sensitivity analysis to determine if there were differences in VL suppression and MTCT between women who received a new HIV diagnosis during pregnancy, women who received a new HIV diagnosis during breastfeeding, and women who received repeat testing during breastfeeding for confirmation of undocumented HIV infection prior to ART (re‐)initiation. Thus, for the sensitivity analysis, we categorized the timing of last positive HIV test as: (1) during pregnancy; (2) new during breastfeeding (i.e. women who newly received a HIV diagnosis during breastfeeding) and (3) repeat during breastfeeding [i.e. women reporting an undocumented HIV diagnosis who require confirmatory testing per national guidelines to start or resume ART [e.g. after return to care or silent transfer to a new facility]].

### 2.5 Outcome ascertainment and definitions

Our main maternal outcome was viral suppression. We defined maternal viral suppression as VL < 1000 copies/mL based on VL results documented in the medical record or from a study‐initiated maternal VL. The main infant outcome was being alive and “HIV undiagnosed” at the time of study data collection. Infant HIV status was based on results of HIV DNA PCR testing (for children >6 weeks but <12 months old) or HIV rapid antibody testing (for children ≥12 months). We also examined the following secondary outcomes: (1) proportion of women with a record of ever receiving ART; (2) proportion of women who started ART at the time of their last positive HIV test; (3) proportion of women who experienced ART interruption since their last pregnancy and (4) proportion of women on ART during the study period.

### 2.6 Covariate definitions and classification

We assessed the distributions of covariates selected for their clinical relevance based on a literature review. We selected the following variables: maternal age in years (16 to 24, 25 to 30, 31 to 46), parity (1, 2 to 3, 4 to 5, >5), marital status (married, single, separated/divorced/widowed), education (never attended school, completed primary, completed secondary, completed tertiary), primary male partner HIV status (negative, positive), travel time to the nearest health facility (<1, 1 to 2, >2 hours), duration participant lived at their current residence (<6, 6 to 12, 13 to 24, >24 months) and receipt of any CFL model service (yes, no). We used directed acyclic graphs (DAGs) to identify confounders of the relationship between the timing of the last positive HIV test and maternal viral suppression, and timing of the last positive HIV test and infant HIV status [14, 15].

### 2.7 Statistical analyses

We summarized clinical and demographic characteristics using proportions and medians, as appropriate. Log‐binomial regression models were used to estimate weighted risk ratios for the association between the timing of the last positive HIV test and failing to achieve viral suppression, and the timing of the last positive HIV test and MTCT. We also used log‐binomial regression models to examine associations between the timing of the last positive HIV test and baseline characteristics and each of our secondary outcomes.

Inverse probability weights (IPW) were used to adjust for confounding. The IPW were defined for each woman as the marginal probability of having a last positive HIV test in the observed time interval (i.e. pregnancy or breastfeeding) divided by the probability of having a last positive HIV test during that time interval conditional on covariates [16]. The numerators and denominators were estimated using logistic regression models that included the following covariates identified as confounders using our DAG: maternal age, parity, marital status, education, travel time to the nearest health facility, duration participant lived at their current residence and availability of CFL models.

All statistical analyses were performed using SAS version 9.4 (SAS, Cary, NC, USA).

## 3 RESULTS

### 3.1 Baseline characteristics

Tracing was initiated for 1633 women in the full cohort and 1058 (64.8%) were successfully contacted. Ninety‐five (9.0%) women contacted did not come to the facility for interviews or declined to participate in the nested cohort because they were not interested, did not have time, or had religious reasons. We reached out to next of kin for 34 (3.2%) women who were dead. Of the 929 women who were screened for enrolment into the nested cohort, 97 (10.4%) were deemed ineligible. A total of 832 women (242 in Lilongwe, 160 in Mzimba North and South, 233 in Salima and 197 in Zomba) enrolled. The median age was 28 years (interquartile range, IQR: 24 to 33) (Table 1). Most women (n = 562, 67.6%) lived ≥1 hour travel distance to the nearest health facility.

**Table 1:** Baseline characteristics of pregnant and breastfeeding women in the study population

Characteristic	N = 832^a^ n (%)
Age	
16 to 24	249 (30.2)
25 to 30	269 (32.7)
31 to 46	306 (37.1)
Parity	
1 child	177 (21.4)
2 to 3 children	340 (41.0)
4 to 5 children	243 (29.3)
>5 children	69 (8.3)
Marital status	
Single	41 (5.0)
Married	587 (71.0)
Separated/divorced/widowed	199 (24.0)
Education level	
No Schooling	66 (7.9)
Primary	493 (59.3)
Secondary	248 (29.9)
Tertiary	24 (2.9)
Primary male partner HIV status	
Negative	212 (37.7)
Positive	337 (60.0)
Indeterminate	1 (0.2)
Don't know	12 (2.1)
Travel distance to the nearest health facility	
<1 hour	269 (32.4)
1 to 2 hour	366 (44.0)
>2 hours	196 (23.6)
Length of time lived at current residence	
<6 months	68 (8.2)
6 to 12 months	87 (10.5)
13 to 24 months	79 (9.5)
>24 months	596 (71.8)
Received any CFL service	
No	77 (9.3)
Yes	749 (90.2)
Don’t know	4 (0.5)
Ever taken ART	
No	43 (5.2)
Yes	789 (94.8)
Started ART at the time of the last positive HIV test	
No	22 (2.6)
Yes	807 (97.4)
ART interruption since last pregnancy	
No	766 (92.3)
Yes	59 (7.1)
No response	3 (0.4)
Don’t know	2 (0.2)
Currently on ART	
No	68 (8.2)
Yes	762 (91.8)

CFL, Community‐facility linkage.

^a^ Categories not adding up to 832 indicate missing data.

### 3.2 Timing of the last positive HIV test

Of 822 women who had the timing of their HIV test documented, 102 (12.4%) had their last positive HIV test during breastfeeding. Living ≥1 hour from the nearest health facility was more common among women who had their last positive HIV test during breastfeeding versus pregnancy. Women who lived one to two hours (PR = 2.15; 95% CI: 1.29 to 3.58) or >2 hours (PR = 2.36; 95% CI: 1.37 to 4.10) travel time to the nearest health facility were more likely to have had their last positive HIV test during breastfeeding compared to women with < 1 hour travel time to the nearest health facility (Table 2). The proportion of women who received CFL model support was similar between women who had their last positive HIV test during breastfeeding (89.1%) versus pregnancy (90.8%) (Table 2).

**Table 2:** Summary of baseline characteristics by the timing of last positive HIV test

	Timing of last positive HIV test		
	Breastfeeding N = 102 n (%)	Pregnancy N = 720 n (%)	Prevalence ratio (95% CI)
Age			
16 to 24	28 (11.5)	216 (88.5)	1.0
25 to 30	31 (11.6)	235 (88.4)	1.02 (0.63 to 1.64)
31 to 46	42 (13.8)	262 (86.2)	1.20 (0.77 to 1.88)
Parity			
1 child	15 (8.7)	157 (91.3)	1.0
2 to 3 children	43 (12.8)	294 (87.2)	1.46 (0.84 to 2.56)
4 to 5 children	33 (13.7)	208 (86.3)	1.57 (0.88 to 2.80)
>5 children	11 (15.9)	58 (84.1)	1.83 (0.88 to 3.78)
Marital status			
Married	70 (12.1)	508 (87.9)	1.0
Separated/Divorced/Widowed	29 (14.6)	170 (85.4)	1.20 (0.80 to 1.80)
Single	3 (7.5)	37 (92.5)	0.62 (0.20 to 1.88)
Education			
No Schooling	14 (21.9)	50 (78.1)	1.0
Primary	67 (13.7)	421 (86.3)	0.63 (0.38 to 1.05)
Secondary	20 (8.2)	225 (91.8)	0.37 (0.20 to 0.70)
Tertiary	1 (4.2)	23 (95.8)	0.19 (0.03 to 1.37)
Primary male partner HIV status			
Negative	19 (9.1)	190 (90.9)	1.0
Positive	42 (12.6)	291 (87.4)	1.39 (0.83 to 2.32)
Travel time to the nearest health facility			
<1 hour	18 (6.8)	247 (93.2)	1.0
1 to 2 hours	53 (14.6)	310 (85.4)	2.15 (1.29 to 3.58)
>2 hours	31 (16.1)	162 (83.9)	2.36 (1.37 to 4.10)
Length of time lived at the current residence			
>24 months	83 (14.1)	507 (85.9)	1.0
13 to 24 months	8 (10.3)	70 (89.7)	0.73 (0.37 to 1.45)
6 to 12 months	3 (3.5)	83 (96.5)	0.25 (0.08 to 0.77)
<6 months	8 (12.1)	58 (87.9)	0.86 (0.44 to 1.70)
Received any CFL service			
Yes	90 (12.2)	649 (87.8)	1.0
No	11 (14.3)	66 (85.7)	1.17 (0.66 to 2.10)

CFL, community‐facility linkage.

There was no statistically significant association between timing of last positive HIV test and the following indicators of ART use: having a record of ever taking ART (aPR = 1.14; 95% CI: 0.47 to 2.75); starting ART at the time of last positive HIV test (aPR = 1.49; 95% CI: 0.49 to 4.53); currently on ART (aPR = 1.28; 95% CI: 0.66 to 2.50) and ART interruption since last pregnancy (aPR = 1.39; 95% CI: 0.70 to 2.77) (Table S1).

### 3.3 Viral load suppression

Eight hundred and nineteen women (98.4%) had VL results available, of whom 111 (13.5%) had unsuppressed VL (i.e. ≥1000 copies/mL). Among women with VL results, 10 women (4 with unsuppressed VL and 6 with suppressed VL) had missing information about the timing of their last positive HIV test. There was no statistically significant difference in the risk of unsuppressed VL between women who had their last positive HIV test during breastfeeding and those who had it during pregnancy (aRR = 0.87; 95% CI: 0.48 to 1.57) (Table 3). In a sensitivity analysis, the risk of unsuppressed VL did not differ significantly between women who had their last positive HIV test during pregnancy and women receiving a new diagnosis during breastfeeding (aRR = 0.47; 95% CI: 0.11 to 2.02) or women receiving repeat testing during breastfeeding (aRR = 1.34; 95% CI: 0.55 to 3.24) (Table 4). Only 11 of 107 (10.3%) women with VL ≥ 1000 copies/mL had their last positive HIV test during breastfeeding.

**Table 3:** Association between the timing of last positive HIV test and maternal and child outcomes

Timing of last positive HIV test	≥1000 copies/mL (N = 107)	<1000 copies/mL (N = 702)	Unweighted risk ratio (95% CI)	IP weighted risk ratio (95% CI)
Viral load				
Pregnancy	96 (13.5)	614 (86.5)	1.0	1.0
Breastfeeding	11 (11.1)	88 (88.9)	0.82 (0.46 to 1.47)	0.87 (0.48 to 1.57)

Timing of last positive HIV test	Yes (N = 31)	No (N = 736)	Unweighted risk Ratio (95% CI)	IP weighted risk ratio (95% CI)
HIV mother‐to‐child transmission				
Pregnancy	17 (2.5)	663 (97.5)	1.0	1.0
Breastfeeding	14 (16.1)	73 (83.9)	6.44 (3.29 to 12.59)	6.57 (3.37 to 12.81)

Confounders for IP weight: age, parity, marital status, education, clinic distance, travel time to the nearest health facility, length of time lived at the same place, and received any community‐facility linkage service. CI, confidence interval; IP, inverse probability.

**Table 4:** Sensitivity analysis – association between timing of last positive HIV test and maternal and child outcomes

Timing of last positive HIV test	≥1000 copies/mL (N = 63)	<1000 copies/mL (N = 682)	Unweighted risk Ratio (95% CI)	IP weighted risk ratio (95% CI)
Viral load				
Pregnancy	58 (8.8)	598 (91.2)	1.0	1.0
Repeat testing during breastfeeding	4 (9.1)	40 (90.9)	1.03 (0.39 to 2.70)	1.34 (0.55 to 3.24)
New diagnosis during breastfeeding	1 (2.2)	44 (97.8)	0.25 (0.04 to 1.77)	0.47 (0.11 to 2.02)

Timing of last positive HIV test	Yes (N = 28)	No (N = 688)	Unweighted risk Ratio (95% CI)	IP weighted risk ratio (95% CI)
HIV mother‐to‐child transmission				
Pregnancy	14 (2.2)	622 (97.8)	1.0	1.0
Repeat testing during breastfeeding	3 (8.1)	34 (91.9)	3.68 (1.11 to 12.25)	2.49 (0.58 to 10.68)
New diagnosis during breastfeeding	11 (25.6)	32 (74.4)	11.62 (5.62 to 24.04)	12.72 (6.31 to 25.62)

Confounders for IP weight: age, parity, marital status, education, clinic distance, travel time to the nearest health facility, length of time lived at the same place, and received any community‐facility linkage service. CI, confidence interval; IP, Inverse probability.

### 3.4 MTCT transmission

By study end, 774 infants had known HIV status, and 32 (4.1%) were HIV positive. Seven mothers of these infants (1 HIV positive and 6 HIV negative) did not have information on the timing of their last positive HIV test. Of the mothers who did, those who had their last positive HIV test during breastfeeding were more likely to experience MTCT (aRR = 6.57; 95% CI: 3.37 to 12.81) compared to mothers who had their last positive HIV test during pregnancy (Table 3). In a sensitivity analysis, the risk of MTCT was higher in women who were newly tested during breastfeeding compared to those who had their last positive test during pregnancy (aRR = 12.72; 95% CI: 6.31 to 25.62) (Table 4).

## 4 DISCUSSION

In this cohort of PBFW living with HIV in the early phase of the “treat all” era in Malawi, over ten percent had their last positive HIV test during breastfeeding. A consequential proportion of these women had unsuppressed VL and experienced MTCT. The risk of unsuppressed VL did not vary with the timing of their last positive HIV test, but MTCT was higher among women who had their last positive HIV test during breastfeeding versus pregnancy.

While the risk of having unsuppressed VL did not vary with the timing of the last positive HIV test, we were unable to ascertain the cumulative time women spent in a viraemic state during the PMTCT cascade since VL is assessed at fixed times beginning at 6 months post‐ART initiation. Nevertheless, high levels of maternal HIV viraemia are known to increase the risk of vertical HIV transmission and pregnancy loss through miscarriage or stillbirth [17, 18, 19, 20, 21]. Unsuppressed VL among PLHIV on ART can be a marker of ART treatment failure or poor adherence [3, 4, 22]. PBFW living with HIV on ART should thus be monitored closely for HIV viraemia and/or treatment failure with serial VL measurement where feasible. Whenever possible, continuous, multi‐disciplinary and client‐centred adherence support should be provided to ensure adequate therapeutic levels of ART to suppress viral replication [23, 24]. Lay health providers delivering CFL models have much to offer in this regard, being able to provide support across multiple behavioural, social and structural domains to promote maternal HIV care engagement and long‐term retention [13].

The MTCT that we observed (4.0%) aligns with Malawi national estimates. During the Malawi Population‐based HIV Impact Assessment (MPHIA) survey (November 2015 to August 2016), 3% of infants under 18 months born to mothers living with HIV were confirmed to be HIV positive [25]. In a cross‐sectional evaluation of outcomes for the Malawian PMTCT programme (October 2014 and May 2016), the MTCT rate at four to twenty‐six weeks was estimated at 4.7% (95% CI: 3.4 to 6.3) [26], similar to estimates from MOH Integrated HIV Program Quarterly Reports [27]. The WHO estimates overall MTCT at the end of breastfeeding to be 9% in Malawi, with 3% and 6% of transmission happening before and after six weeks respectively [28].

The MTCT we observed was driven by mothers who had their HIV status ascertained during breastfeeding. Over 16% of mothers who had their last positive HIV test during breastfeeding experienced MTCT, compared to 2.5% of mothers who had their last positive HIV test during pregnancy. Even though our study found a similar proportion of VL suppression between women who had their last positive test during pregnancy versus breastfeeding, women in the latter group may have gone through pregnancy without ART, started and stopped ART during pregnancy, or seroconverted late in pregnancy or during breastfeeding, thus exposing infants to high viraemia and subsequent risk of MTCT [17, 19, 20].

HIV incidence among PBFW is not rare in sub‐Saharan Africa, with incidence reported as high as 5.37 cases per 100 person‐years [29, 30, 31]. Biological changes and cultural practices put PBFW at higher risk of HIV acquisition. Compared to non‐pregnant women, pregnant women face a higher risk of contracting HIV due to physiological and hormonal changes associated with pregnancy [32, 33, 34]. Some cultural practices in Malawi advise couples to refrain from sexual activity during pregnancy and early breastfeeding periods [35, 36]. During this period, men may seek concurrent sexual partnerships, and, thus, expose women to HIV when sexual activity resumes within the couple. Thus, providing and sustaining pre‐exposure prophylaxis (PrEP) for high‐risk women during pregnancy and breastfeeding is critical, and holds great promise for reducing maternal HIV acquisition and MTCT [37, 38].

In sub‐Saharan Africa, distance to a health facility is a well‐documented barrier not only to HIV testing, but also to healthcare in general. In this study, the majority of women who had their last positive HIV test during breastfeeding lived at least an hour in travel distance away from the nearest health facility. Women who live far away from a health facility have to bear direct and indirect financial costs that affect their ability to seek care, and those on ART are more likely to miss or stop treatment because of these costs [39, 40]. To overcome distance and travel‐related barriers to accessing PMTCT services, new targeted community‐based and differentiated HIV testing modalities can be implemented, such as home‐based couples testing and counselling or HIV self‐testing facilitated by lay health providers [41].

Viral suppression among PLHIV depends on access to an efficacious ART regimen, the duration on ART, and adherence to treatment. In this analysis, we did not take into consideration duration on ART or adherence when estimating viral suppression. If some women in the study had inadequate time on ART (i.e. <8 weeks) to achieve viral suppression, we may have overestimated the true proportion of women with unsuppressed VL. The majority of women in this study reported starting ART at the time of their last positive HIV test. The proportion of women with viral suppression who had their last positive HIV test during pregnancy was surprisingly similar to the proportion who had their last positive HIV test during breastfeeding—a population we would expect to have been on ART for less time compared to women who started ART during pregnancy. Malawi has now adopted dolutegravir‐based therapy in hopes of supporting more rapid and durable VL suppression for PLHIV and of reducing the emergence of HIV resistance. However, we feel that our main findings would likely not be modified by this change in ART regimen. Finally, we did not conduct *a priori* hypothesis testing in this descriptive analysis, and, thus, our finding of no difference in risk of suppressed VL by timing of last positive HIV test may be partly attributed to insufficient statistical power. There is a need for hypothesis‐driven studies to elucidate the association between timing of ART initiation and viral suppression in this population.

In this study, a child’s HIV status was based on available HIV test results within the last 90 days, and when those were not available, study‐initiated test results. At the time of the study, some children were still in follow‐up in the national HEI programme. Hence, children who were HIV‐uninfected during the study should not be interpreted as being “HIV free.” Under Malawian guidelines, a child attains “HIV free survival” status when s/he is ≥24 months old, has a documented negative HIV rapid antibody test, and is no longer breastfeeding [5]. Referring to the proportion of children who were HIV‐uninfected during the study as “HIV free” may over‐estimate true “HIV‐free survival” had we been able to follow all children through age 24 months and breastfeeding cessation.

We used the timing of last positive HIV test as a proxy for the timing of ART initiation to achieve statistical efficiency, which would not have been possible otherwise. The loss in statistical efficiency with a more granular exposure definition is evident from our sensitivity analysis that yielded effect size estimates with wider confidence intervals. However, the use of timing of last positive HIV test as a proxy for ART initiation may have limited the accuracy of our findings and assumes that women initiate ART immediately after HIV diagnosis. Due to sparse data, we could not estimate MTCT for more granular groupings of women who had their last HIV test during breastfeeding, such as those: newly diagnosed as HIV‐positive postpartum with a documented negative or missing test in ANC; known positive but never started ART; and known positive but not on ART during pregnancy. There is a need to examine VL suppression and MTCT, and associated risk factors, in these important sub‐groups through population‐based surveys and prospective cohort studies.

## 5 CONCLUSIONS

Our results show that, in the early treat all era, MTCT occurs disproportionately among mother–infant pairs in which women had their last positive HIV test during breastfeeding, and that timing of the last positive HIV test is associated with structural barriers to HIV services, such as travel distance to the health facility. These findings highlight the need to focus additional resources and differentiated services for breastfeeding women to ensure their access to quality HIV prevention, treatment, care and support along the entire PMTCT continuum.

## COMPETING INTERESTS

No conflict of interest was declared.

## AUTHORS’ CONTRIBUTION

MC, JE, MCH and MEH contributed to study design. MC, JE, LZ and MEH were involved in analysis. MC, JE, LZ, NC, GB, IM, MCH and MEH were involved in the interpretation of the results and manuscript writing.

## Supporting information


**Table S1.** Association between timing of last positive HIV test and indicators of ART use.
