# Supplementary material for: Timing of HIV testing among pregnant and breastfeeding women and risk of mother‐to‐child HIV transmission in Malawi: a sampling‐based cohort study
Source: J Int AIDS Soc. 2021 Mar 21;24(3):e25687. doi: 10.1002/jia2.25687 (PMC7982503; doi:10.1002/jia2.25687)
Supplement: Supplementary file 1 — Table S1. Association between timing of last positive HIV test and indicators of ART use. [file JIA2-24-e25687-s001.docx]

**Supplemental Table 1. Association between timing of last positive HIV test and indicators of ART use.**

| **Record of ever taking ART** | | | | |
| --- | --- | --- | --- | --- |
| Timing of last positive HIV test | No  (N=41) | Yes  (N=781) | Unweighted Prevalence Ratio  (95% CI) | IP Weighted Prevalence Ratio  (95% CI) |
| Pregnancy | 36 (5.0) | 684 (95.0) | 1.0 | 1.0 |
| Breastfeeding | 5 (4.9) | 97 (95.1) | 0.98 (0.39–2.44) | 1.14 (0.47–2.75) |
| **Started ART at the time of HIV ascertainment** | | | | |
| Timing of last positive HIV test | No  (N=22) | Yes  (N=800) | Unweighted Prevalence Ratio  (95% CI) | IP Weighted Prevalence Ratio  (95% CI) |
| Pregnancy | 18 (2.5) | 702 (97.5) | 1.0 | 1.0 |
| Breastfeeding | 4 (3.9) | 98 (96.1) | 1.57 (0.54–4.54) | 1.49 (0.49–4.53) |
| **Currently on ART** | | | | |
| Timing of last positive HIV test | No  (N=65) | Yes  (N=757) N (%) | Unweighted Prevalence Ratio  (95% CI) | IP Weighted Prevalence Ratio  (95% CI) |
| Pregnancy | 54 (7.5) | 666 (92.5) | 1.0 | 1.0 |
| Breastfeeding | 11 (10.8) | 91 (89.2) | 1.44 (0.78–2.66) | 1.28 (0.66–2.50) |
| **Experienced ART interruption since last pregnancy** | | | | |
| Timing of last positive HIV test | Yes  (N=57) | No  (N=760) | Unweighted Prevalence Ratio  (95% CI) | IP Weighted Prevalence Ratio  (95% CI) |
| Pregnancy | 47 (6.6) | 668 (93.4) | 1.0 | 1.0 |
| Breastfeeding | 10 (9.8) | 92 (90.2) | 1.49 (0.78–2.86) | 1.39 (0.70–2.77) |

IP = Inverse probability; CI = Confidence interval

Confounders for IP weight: age, parity, marital status, education, clinic distance, travel time to nearest health facility,

length of time lived at the same place, and received any community-facility linkage service.
